# Dual Workload Related to Agriculture/Fishing and Family Involvement in the Household Among Rural and Small-Scale Fishing Workers in Southern Brazil: Implications for Nursing Care Organization in Primary Health Care

**DOI:** 10.3390/nursrep16070247

**Published:** 2026-07-15

**Authors:** Marta Regina Cezar-Vaz, Clarice Alves Bonow, Shester Cardoso Damaceno, Rudson Amaral da Silva, Geoffrey Obumneme Okoroigwe, Gabriela Laudares Albuquerque de Oliveira

**Affiliations:** 1 School of Nursing, Federal University of Rio Grande, Rio Grande 96203-900, Brazil; obumnemeokoroigwe@gmail.com; 2 Faculty of Nursing, Federal University of Pelotas, Pelotas 96010-610, Brazil; clarice.bonow@ufpel.edu.br (C.A.B.); shester142@gmail.com (S.C.D.); rudson.amaral@gmail.com (R.A.d.S.); gabriela.albuquerque.consultoria@gmail.com (G.L.A.d.O.)

**Keywords:** nursing care, primary health care, workload, rural workers, fishing workers, family

## Abstract

**Background/Objectives**: In rural settings, work, family, and environmental conditions are closely intertwined, requiring Primary Health Care to recognize care needs arising from productive work and everyday family responsibilities. This study aimed to analyze overall and domain-specific levels of perceived workload related to agriculture/fishing and family involvement in the household among rural and small-scale fishing workers, examine associated factors, and discuss analytical implications for nursing care organization in Primary Health Care. **Methods**: This cross-sectional study was conducted in three island territories in Rio Grande, southern Brazil, following the STROBE guidelines. Data were collected using a structured questionnaire on sociodemographic, family, and occupational characteristics and the NASA Task Load Index (NASA-TLX), applied separately to assess workload related to agriculture/fishing and family involvement in the household. **Results**: The sample included 146 rural and small-scale fishing workers. Perceived workload could suggest high in both dimensions, with higher levels in agriculture/fishing (mean 84.0 ± 16.5; 89.7% high/very high) than in family involvement in the household (mean 76.1 ± 24.5; 75.4% high/very high). Agriculture/fishing workload perceived could suggest highest scores for overall effort level, temporal demand, and physical demand, whereas family involvement could suggest highest scores for performance-related workload and overall effort level. In the adjusted model, agriculture/fishing perceived workload may be associated with longer daily working time (b = 1.69; 95% CI: 0.82 to 2.56; *p* < 0.001) and shorter rest time during work (b = −0.05; 95% CI: −0.08 to −0.01; *p* = 0.011). Perceived workload related to family involvement in the household may be associated with monthly income up to two minimum wages (b = 19.90; 95% CI: 7.78 to 32.10; *p* = 0.001). The adjusted R^2^ values were 17.3% and 6.7%, respectively. **Conclusions**: Perceived workload appears to be related to high levels in both productive and family household contexts. The findings might contribute to providing analytical support for future discussions on nursing care organization in Primary Health Care, potentially by considering working conditions, family responsibilities, and territorial context when discussing care needs among rural and small-scale fishing workers.

## 1. Introduction

Rural and small-scale fishing workers often experience work, family life, and environmental conditions as closely connected dimensions of everyday life. In these contexts, productive activities are not easily separated from household organization, family livelihood, care responsibilities, mobility, access to services, and dependence on natural resources. As a result, workload may arise not only from agriculture and fishing, but also from the daily responsibilities required to sustain family life in the household.

This overlap is relevant to Primary Health Care (PHC) because health needs in rural territories are shaped by living and working conditions. A recent explanatory mixed-methods review of PHC systems in seven countries showed that PHC can contribute to universal health coverage and improved health status, although these contributions vary according to how PHC systems are organized and implemented [1]. In Brazil, PHC is structured by the National Primary Care Policy, which emphasizes defined territories, enrolled populations, bonding, accountability, and care coordination [2]. Workers’ health is also addressed by the National Policy on Workers’ Health, which establishes comprehensive care for workers through surveillance, health promotion, protection, and reduction in work-related morbidity and mortality [3].

Rural PHC requires particular attention to the conditions under which people live and work. A scoping review found that rural populations experience worse health outcomes, greater barriers to accessing primary care, and a heterogeneous evidence base for rural PHC interventions [4]. In Brazil, research with Family Health Strategy nurses showed that rural care is shaped by community living conditions, access barriers, and the specific characteristics of work in these territories [5]. These findings suggest that care planning in rural PHC is shaped not only by service availability but also by how work, family life, territory, and access barriers are connected to care needs.

Agriculture and small-scale fishing are especially relevant in this discussion. These activities combine productive work, family labor, subsistence, income, natural resources, and environmental conditions. International frameworks on decent work emphasize workers’ rights, social protection, safer working conditions, and sustainable livelihoods [6,7,8]. The United Nations Decade of Family Farming and the Voluntary Guidelines for Securing Sustainable Small-Scale Fisheries also situate family farming and small-scale fishing within the contexts of food security, community rights, subsistence, and sustainable resource governance [9,10]. Thus, agriculture and fishing should not be understood only as economic activities. They are also forms of work embedded in family life, territory, and socio-environmental conditions.

Nursing plays an important role in addressing these needs in PHC. The World Health Organization emphasizes the central contribution of nurses and midwives to PHC, and the State of the World’s Nursing 2025 report reinforces the essential role of nursing in service delivery, leadership, and health system organization [11,12]. Evidence on family and community nursing also highlights clinical practice, competencies, outcomes, organizational models, and advanced training as relevant domains for care directed toward individuals, families, and communities [13]. However, international models still tend to focus mainly on ill individuals and their families, with less emphasis on prevention, population perspectives, and care organization in complex contexts [14]. Evidence on nurse-led care coordination and interprofessional collaboration also shows that nurses can support continuity, integration, and care planning for people and families with complex needs [15,16,17,18]. Even so, the integration of work, family responsibilities, territory, and environmental conditions remains insufficiently explored in studies of nursing care organization in PHC.

Workload assessment can help to examine this complexity. The NASA Task Load Index (NASA-TLX) is a subjective and multidimensional instrument used to assess perceived workload. Its overall score is based on six domains: mental demand, physical demand, temporal demand, performance, overall effort level, and frustration level [19,20]. This structure allows workload to be examined as a combination of cognitive, physical, temporal, performance-related, effort-related, and affective components. It may therefore be useful for studying populations whose daily lives combine productive work, family responsibilities, and vulnerable living conditions.

Despite this relevance, few studies have examined workload among rural and small-scale fishing workers, considering both agriculture/fishing work and family involvement in the household. This gap is important for PHC and nursing because care needs in these territories may arise from the accumulation of demands across productive work, family life, and access to services. Jointly analyzing these two workload dimensions may provide a broader empirical basis for discussing care planning, prioritization, and longitudinal follow-up in PHC.

Accordingly, this study aimed to analyze overall and domain-specific levels of perceived workload related to agriculture/fishing and family involvement in the household among rural and small-scale fishing workers, to examine associated factors, and to discuss possible implications for the organization of nursing care in Primary Health Care.

## 2. Materials and Methods

### 2.1. Study Design

This is an observational, analytical, cross-sectional study with a quantitative approach, conducted in accordance with the Strengthening the Reporting of Observational Studies in Epidemiology (STROBE) guidelines [21]. The completed STROBE checklist is provided as Supplementary File S1. The cross-sectional design was appropriate for analyzing, at a single point in time, perceived overall workload levels related to agriculture/fishing work and the workload related to family involvement in the household. It also allowed the examination of associations between these outcomes and sociodemographic, occupational, and family characteristics. Given this design, the findings should be interpreted as associative and not causal.

Therefore, two workload outcomes were considered: the perceived workload related to agriculture/fishing, corresponding to the productive work developed in agricultural and/or fishing activities; and the perceived workload related to family involvement in the household, corresponding to the daily responsibilities assumed in the organization, maintenance, and sustenance of family life in the household itself. These outcomes are further detailed below.

### 2.2. Linkage to the Research Project and Study Setting

The results of the present study are part of a larger research project entitled “Nursing in Socio-Environmental Health and the Nature of the Workforce: Studies in Work Processes” (process no. 316678/2023-6), funded under CNPq Call No. 09/2023—Research Productivity Fellowships (PQ–1A), and coordinated at the Federal University of Rio Grande (FURG) by the principal investigator, who is the first author of this manuscript. The larger project is ongoing and includes different research fronts related to the living, working, health, and care conditions of working populations in socio-environmentally vulnerable territories. This manuscript presents a specific cross-sectional analysis of workload related to agriculture/fishing and family involvement in the household among agricultural and/or fishing workers in the selected territories.

This study was conducted in three island territories of the municipality of Rio Grande, in the state of Rio Grande do Sul, Brazil: Ilha dos Marinheiros, Ilha da Torotama, and Ilha do Leonídio. According to municipal records, Ilha dos Marinheiros and Ilha do Leonídio belong to the 2nd District, while Ilha da Torotama is part of the 3rd District (Povo Novo) [22,23]. The selection of these locations is justified by the presence of workers engaged in family farming and artisanal fishing, activities that are relevant to the productive and social organization of these communities [24,25]. Family farming articulates food production, family work, social reproduction, and the management of natural resources [9], while artisanal fishing is related to food security, subsistence, the rights of fishing communities, and the sustainable governance of natural resources [10]. Thus, in these territories, productive work, family life, and local social reproduction develop in close relationship with natural resources, environmental rhythms, conditions of territorial mobility, and access to services [9,10,22,23].

In addition, the three territories present conditions that exacerbate vulnerabilities in both work and everyday life. Recent official records indicate that the islands of Torotama, Marinheiros, and Leonídio are among the areas most susceptible to flooding and inundation in the municipality [26,27]. These events affect housing, mobility, agricultural work, small-scale fishing, and families’ ability to remain in the territory, creating a context of socio-environmental vulnerability and difficult access that is relevant both to understanding the empirical setting and to the operationalization of the study [26,27,28].

### 2.3. Study Population, Eligibility Criteria, and Sampling

The study population consisted of workers living on Ilha dos Marinheiros, Ilha da Torotama, and Ilha do Leonídio who were engaged in family farming and/or small-scale fishing, the predominant activities in the selected territories. The choice of this population was based on the understanding that these workers live and work in territories where productive activities, family life, mobility, access to services, and everyday living conditions are closely shaped by territorial and socio-environmental conditions, as described in the study setting.

The inclusion criteria were as follows: living in one of the selected territories, being at least 18 years of age, and currently working in family farming/horticultural and/or small-scale fishing activities. Eligibility was confirmed by self-report during the face-to-face field approach and interview, based on place of residence, age, and current engagement in the selected activities. Workers who were not engaged in these activities during the data collection period were excluded.

Participants were selected through consecutive convenience sampling between January and February 2026. All eligible and accessible workers identified during the data collection period were invited to participate. A total of 146 agricultural and/or fishing workers completed the study and composed the final analytical sample.

Accurately determining the total number of workers across the three territories was not possible because there was no single, up-to-date, and specific registry of family farming and small-scale fishing workers living on the islands at the time of the study. During fieldwork, households that were closed at the first visit were revisited up to three times on subsequent visits. After these attempts, 567 closed-household records were registered across the three territories. Because eligibility could not be verified in these households, they were not included in the denominator of eligible participants. Among contacted and eligible workers invited to participate, 41 declined after receiving information about the study, and one participant started but did not complete the interview. This incomplete interview was excluded before database closure.

Given these conditions, the accessible population at the time of data collection did not necessarily correspond to the entire population of workers living in these territories. Therefore, given the non-probabilistic sampling strategy and the territorial specificities of the study setting, the findings should be interpreted as context-specific and should not be directly generalized to all agricultural or fishing workers, especially to populations with different employment, ethnic, socioeconomic, or territorial profiles.

### 2.4. Data Collection Procedures

Data collection for this cross-sectional analysis was conducted between January and February 2026 as part of the planned field stage of the larger research project. This period was defined based on the research team’s prior planning, logistical constraints for accessing the island territories, household dispersion, worker mobility, environmental conditions, and fieldwork feasibility.

Data were collected through individual, face-to-face, structured interviews with workers engaged in family farming and/or small-scale fishing. Most interviews were conducted at participants’ homes. When appropriate and feasible, interviews were conducted in work environments, such as fishing sites or agricultural properties. In all situations, privacy, confidentiality, and participant safety were ensured.

The interviews were conducted by pairs of trained interviewers. Training addressed the study objectives, standardized question reading, instrument explanations, ethical procedures, and strategies to reduce interviewer variation. Interviewers were instructed to use accessible language compatible with participants’ level of understanding. When necessary, standardized instructions were repeated or clarified without changing the item content, inducing responses, or interfering with participants’ subjective assessments.

All instruments were interviewer-administered and completed by trained interviewers during face-to-face interviews. At the end of each completed interview, the instruments were checked for completeness by interviewers and under the supervision of the field coordinator and/or the principal investigator. This procedure allowed omitted responses to be identified immediately and, when appropriate, the standardized question was reread to the participant without inducing answers or modifying the content of the items.

Before the interviews, all participants received information about the study objectives and procedures. Participation occurred only after the signing of the Informed Consent Form. Confidentiality, voluntary participation, and the right to withdraw at any time were ensured.

### 2.5. Study Instruments and Variables

Data were collected using two instruments: a structured questionnaire for sociodemographic, family, and occupational characterization and the National Aeronautics and Space Administration Task Load Index (NASA-TLX).

#### 2.5.1. Instrument 1—Sociodemographic and Occupational Questionnaire

The first instrument consisted of a structured questionnaire designed to characterize participants and their living and working contexts. It included sociodemographic, family, and occupational variables.

The sociodemographic and family variables included age, age group, sex, self-reported skin color, marital status, educational level, number of children, number of household members, and monthly income. Occupational variables included occupation, length of employment in agriculture/fishing, weekly working hours, daily working time, rest time during work, work schedule, presence of another job in addition to agriculture/fishing, and employment relationship. Rest time during work was recorded as the cumulative number of minutes of rest reported by the participant on a usual workday, not necessarily as a single continuous break.

Because only one participant self-identified as Black, the Black and Brown categories were combined for statistical comparison purposes. This decision was made to avoid unstable estimates due to the very small number of observations in the Black category. These sociodemographic, family, and occupational variables were considered independent variables potentially associated with the two perceived workload outcomes.

#### 2.5.2. Instrument 2—National Aeronautics and Space Administration Task Load Index

The second instrument was the NASA-TLX, used to assess perceived workload. The NASA-TLX is a subjective, multidimensional instrument composed of six domains: mental demand, physical demand, temporal demand, performance, overall effort level, and frustration level [19,20].

In this study, the NASA-TLX was applied separately to assess two workload dimensions. The first dimension was workload related to agriculture/fishing work, referring to productive activities carried out in family farming and/or small-scale fishing. The second dimension was workload related to family involvement in the household, referring to unpaid daily responsibilities assumed within family life. These responsibilities included household organization and maintenance, food preparation, clothing care, and care for children, older adults, people with disabilities, and animals, among other activities required to sustain everyday family life.

Thus, each participant completed the NASA-TLX twice during the interview: once with reference to agriculture/fishing work and once with reference to family involvement in the household. In both applications, the same six NASA-TLX domains were assessed. Scoring and weighting were performed separately for each workload dimension so that the scores assigned to agriculture/fishing activities were not used to estimate workload related to family involvement, and vice versa.

Mental demand refers to the attention, reasoning, planning, memory, vigilance, and decision-making required to perform an activity. In agriculture/fishing, this included monitoring climatic and environmental conditions, organizing tasks, deciding when and how to perform work, dealing with unforeseen events, and maintaining attention to the production process. Family involvement included planning household tasks, anticipating needs, managing daily time, attending to simultaneous demands from family members, and making decisions related to care and household maintenance.

Physical demand refers to the bodily effort and exertion required. In agriculture/fishing, this included walking, carrying loads, pulling, pushing, lifting, bending, rowing, casting and retrieving nets, handling tools, harvesting, transporting products, and performing repetitive or physically demanding tasks. Family involvement included cleaning, tidying, cooking, washing, organizing the household, transporting items, caring for clothing, assisting dependent individuals, and performing other activities required for daily household maintenance.

Temporal demand refers to time pressure and the pace required to complete activities. In agriculture/fishing, this included rhythms defined by nature and climate, fishing periods, fish preservation, harvesting cycles, production management, and the simultaneous demands of productive and family life. Family involvement reflected the need to perform household tasks and family responsibilities at specific times, continuously, and in coordination with multiple daily demands.

The performance domain refers to the participant’s perception of how successfully the expected activities were accomplished. In the NASA-TLX, this domain has a reverse scoring direction compared with the other domains because lower perceived success represents greater performance-related workload. This was accounted for in the questionnaire scoring procedure by inverting the performance score prior to interpretation. Thus, higher final values in the performance domain indicate greater difficulty in achieving the expected performance and, therefore, higher workload, in the same direction as mental demand, physical demand, temporal demand, overall effort level, and frustration level.

Overall effort refers to the combined mental and physical effort required to perform activities and maintain the level of performance considered necessary by the participant. In agriculture/fishing, it captured physical strain, concentration, persistence, and the need to continue working despite fatigue, adverse environmental conditions, and time or resource constraints. Family involvement refers to the total effort required to sustain family tasks and responsibilities, including household organization, care for individuals, maintenance of the household environment, and responsiveness to the family’s material and relational needs.

Frustration level refers to negative feelings associated with performing activities, such as insecurity, discouragement, irritation, tension, or dissatisfaction. In this study, this domain captured subjective discomfort related to both agriculture/fishing work and family involvement in the household.

Domain scores were analyzed individually, and the overall score was calculated as a weighted mean based on the relative importance assigned by participants to each domain. After weighting, the scores were standardized to a 0–100 scale for interpretation. For descriptive purposes, both overall and domain-specific scores were classified into five fixed-range levels: very low (<20.0), low (20.0–39.9), intermediate (40.0–59.9), high (60.0–79.9), and very high (≥80.0).

During NASA-TLX administration, interviewers ensured that participants understood the reference context for each application: first, agriculture/fishing work, and second, family involvement in the household. When necessary, standardized instructions were reread without changing item content or inducing responses.

The application of the NASA-TLX to family involvement in the household was a contextual use of the instrument to assess perceived workload related to unpaid family responsibilities. It should not be interpreted as a formal content validation of the NASA-TLX for the family domain. Internal consistency was examined separately for both applications in this sample. Cronbach’s alpha was 0.83 for workload related to agriculture/fishing and 0.89 for workload related to family involvement in the household, indicating good internal consistency in both applications.

### 2.6. Statistical Analysis

Workload scores were obtained using the NASA-TLX, as described in the previous section. Each domain was originally scored from 0 to 20. The instrument also includes pairwise comparisons among the six domains, which are used to determine the weight each participant assigns to each domain in calculating the overall score. Thus, the overall score was calculated as a weighted mean, considering the relative importance assigned to the six domains. Domain scores and the overall score were then standardized on a 0–100 scale.

For descriptive purposes, standardized scores were classified into five fixed-width levels of 20 points each: very low (<20.0), low (20.0–39.9), intermediate (40.0–59.9), high (60.0–79.9), and very high (≥80.0), following the logic of equal-width class intervals. This categorization was used only for descriptive interpretation of score magnitude. The multivariable analyses used the continuous overall NASA-TLX scores. Before analysis, data completeness was verified. No missing data were identified for the variables analyzed in the final analytical sample. This was related to the face-to-face, interviewer-administered data collection procedure, in which the instruments were completed by trained interviewers and checked for completeness under supervision. Therefore, all analyses were conducted with 146 participants.

Descriptive analyses were performed to characterize the sample and describe workload scores. Quantitative variables were described using means and standard deviations or medians and interquartile ranges, according to data distribution. Categorical variables were described using absolute and relative frequencies. The distribution of quantitative variables was assessed using the Kolmogorov–Smirnov test, complemented by visual inspection.

Because workload related to agriculture/fishing and workload related to family involvement in the household were measured in the same participants, the two overall scores were compared using the paired-samples *t*-test, with estimation of the mean difference and 95% confidence interval. The Wilcoxon signed-rank test was also used as a complementary non-parametric analysis, considering the concentration of scores in the upper range of the scale.

In bivariate analyses, Student’s *t*-test was used to compare means between two independent groups. Analysis of variance (ANOVA), followed by Tukey’s post hoc test, was used for comparisons among three or more independent groups. Associations between quantitative variables were assessed using Pearson’s correlation coefficient when the distribution was approximately normal, or Spearman’s correlation coefficient when the distribution was skewed.

To control for potential confounding factors, multivariable linear regression was used, with separate models for the two outcomes: workload related to agriculture/fishing and workload related to family involvement in the household. Variables with *p* < 0.20 in the bivariate analysis were considered for entry into the initial models.

For workload related to agriculture/fishing, the initial model included self-reported skin color/race, educational level, monthly income, occupation, length of employment in agriculture/fishing, weekly working hours, daily working time, and rest time during work. For workload related to family involvement in the household, the initial model included self-reported skin color/race, monthly income, occupation, length of employment in agriculture/fishing, daily working time, rest time during work, and having another job in addition to agriculture/fishing. Categorical variables with more than two categories were entered using dummy variables.

Although employment relationship should be interpreted as statistically significant in the bivariate analysis for both outcomes, this variable was not entered into the main multivariable models because of its highly asymmetric distribution in the sample, with only four formally employed workers. This decision was adopted to avoid possible unstable and imprecise estimates. Thus, employment relationship was retained only as a descriptive and contextual variable. Supplementary Table S1 presents the candidate variables entered into the multivariable models and indicates whether each variable was retained or excluded after the backward selection procedure.

Final models were obtained using the backward method. Variables with *p* < 0.10 were retained in the final models. Unstandardized regression coefficients (b), with their respective 95% confidence intervals, and standardized beta coefficients (β) were estimated. Standardized beta coefficients were used to compare the magnitude of associations among the variables retained in each model. The coefficient of determination (R^2^) and adjusted R^2^ were calculated to describe the proportion of variability explained by each model. Statistical significance was interpreted at *p* ≤ 0.05. Analyses were performed using IBM SPSS Statistics, version 27.0. Multicollinearity among the independent variables was assessed using tolerance and variance inflation factor (VIF). Following the criteria proposed by Hair et al. (2019) [29], VIF values <5.0 and tolerance values >0.20 were adopted to indicate the absence of problematic multicollinearity and to support the stability of the regression coefficients. The adequacy of the models and the assumption of homoscedasticity were assessed through visual inspection of residual plots, specifically residuals versus predicted values. The residual plots showed a random distribution of points, without clear patterns or trends, supporting the adequacy of the regression assumptions.

The concentration of participants in the high and very high workload categories was examined descriptively to assess the possibility of a ceiling effect and its implications for score discrimination in the upper range of the scale. Because of the cross-sectional design and the two-month data collection period, the analyses do not allow causal inference or assessment of seasonal variations in workload related to agriculture/fishing or family involvement in the household.

### 2.7. Interpretive Procedure for Discussing the Implications of the Findings for the Organization of Nursing Care in Primary Health Care

After the quantitative analyses, an interpretive procedure was defined to guide the discussion of the possible implications of the findings for the organization of nursing care in Primary Health Care. This procedure did not constitute a new empirical phase, an intervention study, the formal development of a care model, or the validation of a care model. Its purpose was to delimit the interpretation of the quantitative results, considering the cross-sectional design, the characteristics of the sample, and the construct of perceived workload assessed by the NASA-TLX.

The interpretation considered four articulated elements. The first element was the two workload dimensions assessed: workload related to agriculture/fishing and workload related to family involvement in the household. These two dimensions were examined using the NASA-TLX domains for each application [19,20]. The second element was the set of associations identified in the statistical models, understood as associative, non-causal, and context-dependent findings. The third element was the empirical context of rural and small-scale fishing workers living in island territories with socio-environmental vulnerabilities. This empirical context also included the sociodemographic, family, and occupational profile of the sample, such as age distribution, sex, self-reported skin color/race, educational level, income, occupation, and working-time characteristics. These characteristics were considered contextual descriptors of the studied population, not evidence of statistical association, unless supported by the analytical models. The fourth element was the conceptual and guiding frameworks used in the manuscript to discuss nursing care in Primary Health Care.

The frameworks used included Primary Health Care, due to its orientation toward comprehensive, continuous care delivered close to people’s everyday life contexts [13,14,16]; workers’ health, from the perspective of surveillance, health promotion, protection, and comprehensive care for workers [3]; and family and community nursing, because of its understanding of care directed toward individuals, families, and communities within their life contexts [13,14]. Nurse-led care coordination and interprofessional collaboration were considered due to their relevance to the organization of follow-up and continuity of care in complex situations [15,16,17,18]. In addition, the frameworks for decent and fair work [6,7,8] and international documents on family farming and small-scale fishing [9,10] were considered for their relevance to work, family livelihood, territory, and socio-environmental sustainability.

**Figure 1:**
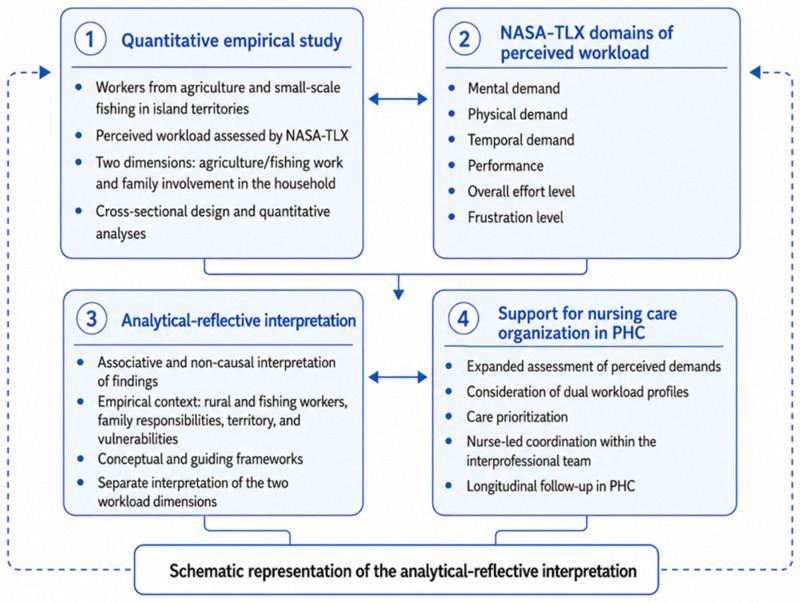
Outline of the analytical–reflective stage developed after the quantitative analyses. The figure summarizes how empirical findings, the two workload dimensions assessed through separate NASA-TLX applications, and the study’s conceptual and guiding frameworks informed analytical implications for nursing care organization in Primary Health Care. This represents an interpretive synthesis and not a new empirical or interventional phase.

The two NASA-TLX applications were interpreted separately to preserve the distinction between perceived workload in productive work and perceived workload in everyday family responsibilities. Figure 1 schematically summarizes the interpretive procedure used to relate the quantitative empirical study to the dimensions of perceived workload, the analytical–reflective interpretation of the findings, and the potential to support nursing care organization in Primary Health Care.

The analytical implications presented in the Discussion were not assessed for feasibility or acceptability with Primary Health Care nurses, workers, communities, or interprofessional teams. Therefore, they should be understood as analytical support for future discussions, care planning, and research, rather than as recommendations for immediate implementation. Further longitudinal, implementation, or intervention studies are needed to examine whether and how these implications may contribute to context-sensitive care strategies in Primary Health Care.

### 2.8. Ethical Aspects

This study was conducted in accordance with the ethical principles of the Declaration of Helsinki [30] and with the Brazilian ethical regulations for research involving human participants, as established by National Health Council Resolution No. 466/2012 [31]. It was approved by the Research Ethics Committee of the Federal University of Rio Grande (FURG) under CAAE No. 93425425.0.0000.5324, approval No. 8.030.097, on 4 December 2025. All participants were informed about the study objectives, procedures, risks, benefits, confidentiality, and the voluntary nature of their participation. Written informed consent was obtained from all participants before data collection.

## 3. Results

The sample consisted of 146 agricultural and/or fishing workers. The mean age was 56.9 years (±13.7 years). The 40–59 age group was the largest (47.9%), while older adults accounted for a considerable proportion of the sample (40.4%). Most participants were male (70.5%) and self-identified as White (87.7%). Because only one participant self-identified as Black (0.7%), the Black and Brown categories were combined for statistical comparison.

Regarding sociodemographic and occupational characteristics, most participants had a partner (70.5%), had completed up to elementary education (81.5%), had a monthly income of up to two minimum wages (88.4%), and were self-employed (97.3%). The majority were engaged in fishing (70.5%), followed by agriculture (17.1%) and both activities (12.3%). Seven participants (4.8%) reported having additional employment alongside agriculture or fishing. Working hours were intensive, with a mean of 14.3 h per day, a median of 120 min of rest during the workday, and an average of 78.1 h per week.

Considering the two outcomes analyzed, workload related to agriculture/fishing corresponded to the productive, remunerated labor dimension, whereas workload related to family involvement corresponded to the daily activities and responsibilities within family life dimension.

The mean overall score for perceived workload in the agriculture/fishing dimension was 84.0 points (±16.5), and the median was 87 points. While the mean perceived workload for the family involvement dimension was 76.1 points (±24.5), the median was 84 points. Regarding the distribution across levels, the high and very high categories accounted for 89.7% of the sample in agriculture/fishing-related workload and 75.4% in family-related workload, which could suggest elevated levels of workload in both dimensions analyzed (Table 1).

**Figure 2:**
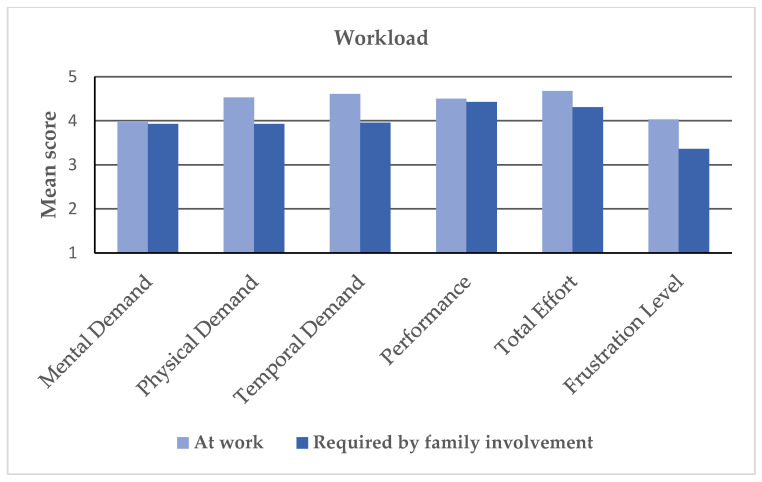
Representation of perceived workload score for agriculture/fishing work and family involvement in the household. Mean NASA-TLX scores, originally standardized on a 0–100 scale, were mapped to five categorical levels for graphical presentation: 1 = very low (<20.0), 2 = low (20.0–39.9), 3 = intermediate (40.0–59.9), 4 = high (60.0–79.9), and 5 = very high (≥80.0).

In the analysis of NASA-TLX domains related to agriculture/fishing work, the highest mean scores should be observed for overall effort level (89.8 ± 18.5), temporal demand (86.5 ± 21.4), and physical demand (86.1 ± 22.2), regardless of the measure of central tendency, with more than 85% of the sample showing high or very high levels in these three factors. Regarding workload related to family involvement, the highest mean scores should be observed for performance (82.8 ± 22.2) and overall effort level (80.8 ± 29.4), regardless of whether the mean or median was used; about 80% of the sample appears to be at the highest workload level. Figure 2 illustrates the distribution of the six domains across the two workload dimensions.

The paired comparison of overall workload levels: in general, workload appears to be higher in agriculture/fishing than in family involvement (except for mental demands and performance); details are shown in Table 2.

In the bivariate analyses, several variables should be interpreted as statistically significant associations with each workload dimension. For workload related to agriculture/fishing, associations appears to be related to with monthly income of up to two minimum wages (*p* = 0.010), occupation as a fisher compared with farmers (*p* = 0.037), longer weekly working hours (*p* = 0.032), shorter rest periods during work (*p* < 0.001), longer daily working hours (*p* < 0.001), and self-employment status (*p* < 0.001). For workload related to family involvement, associations appears to be related to with monthly income of up to two minimum wages (*p* = 0.003), concurrent engagement in both farming and fishing compared with farming alone (*p* = 0.029), shorter rest periods during work (*p* = 0.019), longer daily working hours (*p* = 0.033), and self-employment status (*p* = 0.013). The details of the potential of these associations are detailed in Table 3.

To control for potential confounding factors, variables with *p* < 0.20 in the bivariate analysis were considered for the multivariable linear regression models. Employment relationship could suggest as a statistically significant variable in the bivariate analysis for both outcomes. However, this variable was not included in the main multivariable models because only 4 participants were formally employed, which could yield unstable and imprecise estimates. The complete set of candidate variables included in the multivariable models, along with their retention or exclusion after backward selection, is presented in Supplementary Table S1. The final models retained variables with *p* < 0.10 and are shown in Table 4.

After adjustment, the model for perceived workload related to agriculture/fishing may be associated statistically with daily working time (*p* < 0.001) and rest time during work (*p* = 0.011). Monthly income up to two minimum wages and self-reported White skin color/race remained in the model according to the adopted retention criterion, but did not reach statistical significance at the 5% level (*p* = 0.081 and *p* = 0.062, respectively). This model explained 19.6% of the variability in overall workload related to agriculture/fishing, with an adjusted R^2^ of 17.3%. Therefore, it is necessary to be cautious in interpreting these associations.

For each additional hour of daily working time, there was a mean increase of 1.69 points in overall workload related to agriculture/fishing (95% CI: 0.82 to 2.56). Conversely, for each additional minute of rest time during work, there was a mean reduction of 0.05 points in this outcome (95% CI: −0.08 to −0.01). Based on the standardized regression coefficient (β), daily working time may be shown to have the strongest association in this model.

In the model for workload related to family involvement in the household, monthly income up to two minimum wages appears to remain statistically associated with higher workload (*p* = 0.001). Self-reported White skin color/race remained in the model according to the adopted retention criterion, but did not reach statistical significance at the 5% level (*p* = 0.076). This model explained 8.0% of the variability in workload related to family involvement in the household, with an adjusted R^2^ of 6.7%. In this analytical context, therefore, the interpretations should be viewed with caution regarding possible associations.

The findings could suggest workers with a monthly income of up to two minimum wages had, on average, a 19.9-point higher workload related to family involvement in the household than workers with higher incomes (95% CI: 7.78 to 32.1). Based on the standardized regression coefficient (β), monthly income may represent the strongest association in this model.

Overall, the findings appear related to an association with high perceived workload across both workload dimensions, with higher scores for agriculture/fishing than for family involvement in the household. The adjusted models could suggest that workload related to agriculture/fishing could be interpreted as mainly associated with the organization of working time, particularly longer daily working time and shorter rest time during work. In contrast, workload related to family involvement in the household could be interpreted as mainly associated with lower monthly income. These models accounted for a limited proportion of variability in both outcomes and should therefore be interpreted as partial and context-dependent explanations of perceived workload.

## 4. Discussion

The findings of this study may be associated with high perceived workload in both workload dimensions, with higher scores for agriculture/fishing than for family involvement in the household. In the adjusted models, workload related to agriculture/fishing appears to be related to daily working time and rest time during work, whereas workload related to family involvement in the household appears to be mainly associated with monthly income. These findings should be interpreted as an association, context-specific, and non-causal, considering the cross-sectional design, the concentration of scores in the upper range of the scale, and the limited explanatory power of the models.

### 4.1. Workload Levels Related to Agriculture/Fishing and Family Involvement in the Household

The findings may be associated with high perceived workload across both assessed dimensions, with higher scores for agriculture/fishing than for family involvement in the household. This difference appears to be related to the physical, temporal, environmental, and organizational demands involved in family farming and small-scale fishing [4,9,10]. These activities are known as often connected to natural resources, climatic conditions, production cycles, mobility, and access to services, which may shape perceived workload in rural and fishing territories [22,23,24,25].

The high scores observed for family involvement could also suggest that unpaid household responsibilities could contribute to the perceived workload. This finding should be interpreted with caution as an association; however, it is relevant because family farming and small-scale fishing extend beyond productive activities. They are also connected to family life, subsistence, social reproduction, and territorial conditions [9,10]. In this sense, family involvement should be interpreted as an important component of everyday workload in contexts where productive work, household responsibilities, and territorial vulnerabilities are closely interconnected.

These findings are also aligned with evidence suggesting that rural Primary Health Care needs to consider the living and working conditions of populations in their territories [4,5]. From a nursing perspective, this is relevant because family and community nursing emphasizes care directed toward individuals, families, and communities within their life contexts [13,14]. In the present study, the dual assessment of perceived workload might help make visible that care needs are not only related to occupational activities but might also be linked to the accumulation of daily responsibilities within the household. This interpretation is limited because the study did not measure the specific type, intensity, or distribution of family responsibilities among household members.

The concentration of scores in the upper range of the NASA-TLX scale should also be considered. This pattern appears to reflect genuinely high perceived workload in the studied territories. However, it may also indicate reduced discrimination among participants with very high workload or a possible response ceiling effect. For this reason, differences within the high and very high perceived workload range should be interpreted with caution, especially when comparing workload levels between the two dimensions.

### 4.2. Factors Associated with Workload in the Adjusted Models

In the adjusted model for workload related to agriculture/fishing, daily working time and rest time during work may be associated with the outcome. Longer daily working time may be associated with higher perceived workload, whereas longer rest time during work may be associated with lower perceived workload. These findings could suggest that the organization of working time may be relevant to understanding workload in family farming and small-scale fishing. However, the association with rest time should be interpreted with caution because the coefficient magnitude was small.

The statistics’ relationship with daily working time appears to be related to the nature of agricultural and fishing activities, which often depend on production cycles, climatic conditions, environmental rhythms, mobility, and access to natural resources [9,10,22,23,24,25]. In these contexts, working time may not be organized only by formal schedules. It may also be shaped by weather, harvesting periods, fishing dynamics, product preservation, transport, and the need to reconcile productive and family responsibilities. Therefore, longer daily working time may reflect a broader organization of everyday labor rather than only the number of hours worked.

Rest time during work also requires careful interpretation. Although longer rest time appears to be associated with lower workload, the coefficient indicates a small mean reduction in the workload score for each additional minute of rest. This result should not be interpreted as evidence that increasing rest time alone would substantially reduce workload. Rather, it could suggest that opportunities for rest may be one component of how workers experience and manage workload in daily activities. This interpretation appears to be consistent with broader principles of decent work, which emphasize safer working conditions, social protection, and the organization of work in ways that support workers’ health and well-being [6,7,8].

In the model for perceived workload related to family involvement in the household, monthly income up to two minimum wages may be statistically associated with higher perceived workload. This finding should be interpreted as indicating that lower income may be associated with a greater perceived burden of unpaid family responsibilities. In households with fewer economic resources, daily family organization may require greater effort to manage food, care, household maintenance, transport, and access to services. Nevertheless, this interpretation should be made with caution because the study did not measure the specific composition of family responsibilities, the number of dependents requiring care, or the distribution of unpaid work among household members.

Self-reported White skin color/race remained in both final models according to the adopted retention criterion, but did not reach statistical significance at the 5% level. Therefore, this variable should not be interpreted as demonstrating a statistically significant association with workload in the adjusted models. It may be considered only as part of the contextual description of the sample and as an indicator that future studies with larger and more diverse populations could examine how racial/ethnic, socioeconomic, and territorial inequalities may intersect with workload in rural and fishing contexts. Employment relationship was not included in the main adjusted models because only four participants were formally employed. This analytical decision reduced the risk of unstable and imprecise estimates. Therefore, employment relationship may not be interpreted as an adjusted factor associated with workload. It remained only a contextual characteristic of the studied population, suggesting it was predominantly self-employed.

The explanatory power of the adjusted models appeared limited. The model for agriculture/fishing explained 19.6% of the variability in perceived workload, whereas the model for family involvement in the household explained 8.0%. These values indicate that the models captured only part of the variability in the outcomes. Other factors not measured in this study may contribute to perceived workload, including health conditions, pain, fatigue, sleep, social support, household composition, dependent family members, access to services, environmental stressors, and seasonal variations. Therefore, the adjusted models should be interpreted as partial and context-dependent explanations of perceived workload, not as predictive tools for care decision-making.

### 4.3. Analytical Implications for Nursing Care Organization in Primary Health Care

Based on the interpretive procedure described in the Methods section, the findings provide analytical support for discussing possible implications for nursing care organization in Primary Health Care. These implications should not be understood as a validated care model, an intervention protocol, or recommendations for immediate implementation. In this sense, the potential contribution of the study lies in discussing how perceived workload may inform future discussions about care needs among rural and small-scale fishing workers, especially when productive work, family responsibilities, and territorial conditions are considered together.

The first potential analytical implication concerns the expanded assessment of perceived workload demands by farmers and fishermen in nursing care. The high workload scores may be associated with both assessed dimensions, suggesting that perceived workload demands, although not always expressed as clinical complaints, could be considered in nursing assessments within Primary Health Care. In rural and fishing territories, these demands may include daily working time, rest opportunities, physical strain, temporal pressure, income constraints, family responsibilities, household organization, care-related tasks, access barriers, and environmental conditions. This perspective appears to be consistent with Primary Health Care principles, which emphasize comprehensive, continuous, and context-sensitive care [1,2,3,4,5], and with workers’ health approaches, which situate work as a central dimension of surveillance, protection, health promotion, and comprehensive care [3]. It also suggests demonstrating coherence with family and community nursing perspectives, which emphasize care directed toward individuals, families, and communities within their life contexts [13,14].

The second analytical implication concerns the consideration of dual perceived workload profiles. The findings suggest that workload related to agriculture/fishing and workload related to family involvement in the household appear to be both high, but differ in magnitude, predominant NASA-TLX domains, and associated factors. Considering these two dimensions together may support future discussions about whether perceived workload is more strongly related to productive work, family responsibilities, or the simultaneous accumulation of demands in both spheres. This does not imply a formal risk classification validated by the present study. Rather, it might contribute to offering an analytical basis for discussing how care needs may be identified, followed, and prioritized in Primary Health Care, especially in populations whose work, family life, and territory are closely connected.

The third analytical implication refers to nurse-led coordination within the interprofessional team. International evidence highlights the role of nurses in service delivery, leadership, care coordination, and health system organization [11,12]. Evidence on family and community nursing also indicates that nursing can contribute to continuity of care and to responses directed toward individuals, families, and communities within their life contexts [13,14]. In addition, studies on nurse-led care coordination and interprofessional collaboration in Primary Health Care emphasize continuity, communication, shared objectives, role clarity, and coordination among professionals and services [15,16,17,18]. In the context of the present study, these references support the discussion that nurses may be strategically positioned to identify workload-related care needs, discuss priorities with workers and families, and articulate support with community health workers and other professionals. However, these possibilities require future evaluation with Primary Health Care teams, workers, families, and communities.

The fourth analytical implication concerns longitudinal follow-up. The high perceived workload across both dimensions may be linked to daily working time, rest time during work, and income, suggesting that workload may be linked to persistent conditions of everyday life rather than only to isolated episodes of illness or acute complaints. Longitudinal follow-up may suggest that Primary Health Care teams reassess workload over time and observe changes related to productive cycles, family composition, health status, access to services, environmental conditions, and seasonal variation. This is particularly relevant in rural and fishing territories, where agricultural and fishing activities are closely connected to family livelihood [32,33], natural resources [9,10], territorial mobility, occupational risks, seasonality, climatic conditions [34,35], and socio-environmental vulnerabilities [36,37,38].

Interprofessional collaboration may be relevant for translating these analytical elements into future discussions on care organization. Reviews on interprofessional collaboration in Primary Health Care indicate that effectiveness depends on role clarity, communication, shared objectives, and organizational conditions that support collaborative practice [17,18]. In rural and fishing territories, nurses might contribute to identifying and monitoring workload-related needs, while community health workers may support territorial knowledge, home follow-up, and recognition of changes in daily routines. Depending on local availability, other professionals might contribute to the assessment of clinical conditions, functional limitations, mental health, food organization, social protection, and access to rights. These possibilities should be understood as areas for future exploration, not as care pathways tested or validated by the present study.

Taken together, these potential analytical implications might collaboratively reinforce the relevance of discussing nursing care in Primary Health Care in relation to the concrete conditions of life and work experienced by rural and small-scale fishing workers. Workload assessment might contribute to future discussions on care priorities, longitudinal follow-up, and context-sensitive care strategies. The present study did not intend to evaluate the feasibility, acceptability, and effectiveness of incorporating this type of assessment into routine Primary Health Care practice. Further longitudinal, qualitative, mixed-methods, implementation, or intervention studies may be carried out to examine whether and how dual workload assessment can contribute to care planning, follow-up, and interprofessional work in rural and fishing territories.

### 4.4. Limitations, Strengths, and Future Research

The findings of this study should be interpreted considering several limitations. First, the cross-sectional and observational design does not allow causal inference, but only the identification of associations between perceived workload and the variables analyzed. Second, the use of consecutive convenience sampling and the specific territorial context of the three island communities limit the generalizability of the findings to other rural, agricultural, or fishing populations and care settings. Third, the data collection period was restricted to two months and did not allow assessment of seasonal variations in agricultural and fishing activities. This is relevant because productive cycles, climatic conditions, environmental stressors, flooding events, and access barriers may be associated with variations in workload over time, especially in territories where work is closely related to natural resources and environmental rhythms [9,10,34,35].

Fourth, the concentration of participants in the high and very high workload categories suggests a possible ceiling effect, which may have limited score variability, and reduced the ability to identify differences between subgroups. Fifth, this study did not measure some variables that may be related to perceived workload, such as social support, health conditions, the presence of dependent family members, the distribution of household responsibilities, psychological distress, sleep quality, pain, functional limitations, and access to social protection. The study also did not examine in depth the gendered distribution of productive and family responsibilities. These aspects may be particularly relevant for interpreting workload related to family involvement in the household, for which the adjusted model accounted for a smaller proportion of variability. Therefore, the associations identified should be understood as partial and context-dependent markers of workload, rather than as exhaustive explanations of the phenomenon.

Sixth, the application of the NASA-TLX to family involvement in the household was a contextual use of the instrument. Although internal consistency was good in both applications, this should not be interpreted as a complete psychometric validation of the NASA-TLX for the family involvement dimension. Finally, the potential analytical implications developed from the empirical findings were not assessed a priori for feasibility or acceptability with Primary Health Care nurses, workers, communities, or interprofessional teams. The return of the findings will be carried out in accordance with the researchers’ responsibility agreement with Primary Health Care services, the communities, and the Municipal Health Department of Rio Grande.

Despite these limitations, this study has important strengths. It used an internationally recognized multidimensional instrument to assess perceived workload and applied the NASA-TLX separately to two workload dimensions experienced by the same participants: agriculture/fishing work and family involvement in the household. This approach appears to have allowed the study to distinguish not only the magnitude of workload, but also the predominant domains through which workload was expressed in each dimension. In addition, the study addressed a population and territorial context that remain underrepresented in workload and nursing care research, which may contribute evidence relevant to Primary Health Care, workers’ health, and family and community nursing.

Future studies could use longitudinal designs to examine seasonal and environmental variations in workload and include larger and more diverse samples from different rural and fishing contexts. Such studies could also consider sufficient variability in labor arrangements when these characteristics are analytically relevant. Qualitative or mixed-methods studies could deepen understanding of how workers and families perceive, negotiate, and distribute productive and family responsibilities in everyday life. In addition, implementation or intervention studies could examine how dialogue among workers, communities, and Primary Health Care professionals may help determine whether dual workload assessment can contribute to discussions about care needs and context-sensitive care strategies.

## 5. Conclusions

In this context-specific sample, high levels of perceived workload may be associated with both assessed dimensions: agriculture/fishing work and family involvement in the household. Although perceived workload may be higher in agriculture/fishing, family involvement also showed elevated scores, suggesting that perceived burden could be distributed across productive and family spheres of everyday life. The two workload dimensions differed in their predominant NASA-TLX domains and associated factors. Workload related to agriculture/fishing may be more closely associated with the organization of working time, whereas workload related to family involvement in the household appears to be more closely associated with income.

These findings may provide analytical support for future discussions on nursing care organization in Primary Health Care. They may suggest the relevance of considering working conditions, family responsibilities, and territorial context when discussing care needs among rural and small-scale fishing workers. However, these analytical implications should be interpreted with caution, given the cross-sectional design, the contextual nature of the sample, the possible ceiling effect, and the limited explanatory power of the adjusted models.

The findings may also suggest the need for longitudinal studies, continued follow-up of these workers, and the return of results to the communities and Primary Health Care professionals. Such a process may inform future discussions on how working conditions, family life, and territorial context could be considered in context-sensitive care strategies.

## Figures and Tables

**Table 1:** Overall and domain-specific levels of workload related to agriculture/fishing and related to family involvement in the household among rural and fishing workers (n = 146).

	Workload Related to Agriculture/Fishing						
Domains	Mean ± SD	Median (P25–P75)	Very Low (<20%)	Low (20–39.9%)	Intermediate (40–59.9%)	High (60–79.9%)	Very High (≥80%)
			n (%)	n (%)	n (%)	n (%)	n (%)
Mental Demand	71.5 ± 29.4	75 (50–100)	12 (8.2)	6 (4.1)	29 (19.9)	27 (18.5)	72 (49.3)
Physical Demand	86.1 ± 22.2	100 (75–100)	3 (2.1)	4 (2.7)	13 (8.9)	18 (12.3)	108 (74.0)
Temporal Demand	86.5 ± 21.4	100 (80–100)	3 (2.1)	3 (2.1)	10 (6.8)	16 (11.0)	114 (78.1)
Performance	84.8 ± 20.8	95 (75–100)	2 (1.4)	0 (0.0)	20 (13.7)	25 (17.1)	99 (67.8)
Overall Effort Level	89.8 ± 18.5	100 (90–100)	1 (0.7)	2 (1.4)	14 (9.6)	8 (5.5)	121 (82.9)
Frustration Level	74.2 ± 31.4	90 (50–100)	16 (11.0)	5 (3.4)	25 (17.1)	13 (8.9)	87 (59.6)
Total	84.0 ± 16.5	87 (76–98)	0 (0.0)	3 (2.1)	12 (8.2)	32 (21.9)	99 (67.8)
	**Workload related to family involvement in the household**						
Mental Demand	71.7 ± 33.7	80 (50–100)	22 (15.1)	2 (1.4)	19 (13.0)	24 (16.4)	79 (54.1)
Physical Demand	72.9 ± 36.0	90 (50–100)	26 (17.8)	1 (0.7)	20 (13.7)	9 (6.2)	90 (61.6)
Temporal Demand	73.0 ± 35.7	90 (50–100)	24 (16.4)	4 (2.7)	17 (11.6)	10 (6.8)	91 (62.3)
Performance	82.8 ± 22.2	95 (74–100)	2 (1.4)	3 (2.1)	24 (16.4)	18 (12.3)	99 (67.8)
Overall Effort Level	80.8 ± 29.4	95 (75–100)	13 (8.9)	3 (2.1)	14 (9.6)	12 (8.2)	104 (71.2)
Frustration Level	59.5 ± 37.8	85 (50–100)	32 (21.9)	20 (13.7)	18 (12.3)	15 (10.3)	61 (41.8)
Total	76.1 ± 24.5	84 (61–99)	2 (1.4)	18 (12.3)	16 (11.0)	28 (19.2)	82 (56.2)

**Table 2:** Paired comparison of overall levels of dual workload, related to agriculture/fishing and family involvement in the household, between rural workers and fishermen (n = 146).

Domains	Workload Related to Agriculture/Fishing Mean ± SD	Workload Related to Family Involvement in the Household Mean ± SD	*p*
Mental Demand	71.5 ± 29.4	71.7 ± 33.7	0.959
Physical Demand	86.1 ± 22.2	72.9 ± 36.0	<0.001
Temporal Demand	86.5 ± 21.4	73.0 ± 35.7	<0.001
Performance	84.8 ± 20.8	82.8 ± 22.2	0.268
Overall Effort Level	89.8 ± 18.5	80.8 ± 29.4	<0.001
Frustration Level	74.2 ± 31.4	59.5 ± 37.8	<0.001
Total	84.0 ± 16.5	76.1 ± 24.5	<0.001

**Table 3:** Bivariate associations between participants’ characteristics and workload related to agriculture/fishing and family involvement in the household (n = 146).

Variables	n (%)	Agriculture/Fishing Workload	*p*	Family Involvement Workload	*p*
		Mean ± SD		Mean ± SD	
Age (years) *	56.9 ± 13.7	r = −0.081	0.330 ^a^	r = −0.058	0.484 ^a^
Age group			0.247 ^b^		0.587 ^b^
<40 years	17 (11.6)	85.7 ± 9.30		80.0 ± 13.7	
40–59 years	70 (47.9)	85.9 ± 15.6		77.0 ± 26.1	
≥60 Years	59 (40.4)	81.2 ± 18.8		73.8 ± 25.0	
Sex			0.870 ^c^		0.332 ^c^
Male	103 (70.5)	83.9 ± 16.8		74.8 ± 25.0	
Female	43 (29.5)	84.4 ± 16.1		79.1 ± 23.2	
Skin color			0.124 ^c^		0.189 ^c^
White	128 (87.7)	85.0 ± 15.8		77.1 ± 24.4	
Black/Brown	18 (12.3)	77.1 ± 20.0		68.9 ± 24.8	
Has a partner			0.253 ^c^		0.245 ^c^
Yes	103 (70.5)	85.1 ± 15.3		77.6 ± 23.9	
No	43 (29.5)	81.3 ± 19.1		72.4 ± 25.8	
Educational level			0.140 ^c^		0.679 ^c^
Up to completed elementary school	119 (81.5)	85.0 ± 16.5		75.7 ± 25.5	
Incomplete high school or higher	27 (18.5)	79.8 ± 16.1		77.6 ± 20.0	
Number of children **	2 (1–3)	r_s_ = −0.066	0.426 ^d^	r_s_ = −0.033	0.690 ^d^
Number of household members **	2 (2–3)	r_s_ = 0.008	0.923 ^d^	r_s_ = −0.021	0.799 ^d^
Monthly income (minimum wages) *			0.010 ^c^		0.003 ^c^
Up to 2	129 (88.4)	85.3 ± 15.6		78.2 ± 23.8	
More than 2	17 (11.6)	74.3 ± 20.2		59.7 ± 23.8	
Occupation			0.037 ^b^		0.029 ^b^
Farmer	25 (17.1)	76.6 ± 19.8		65.3 ± 25.4	
Fisher	103 (70.5)	86.0 ± 15.4 ^#^		77.3 ± 24.6	
Both	18 (12.3)	82.9 ± 15.5		84.1 ± 17.7 ^#^	
Length of employment in agriculture/fishing (years) *	39.0 ± 16.3	r = 0.157	0.059 ^a^	r = 0.118	0.157 ^a^
Weekly working hours *	78.1 ± 22.0	r = 0.178	0.032 ^a^	r = −0.044	0.600 ^a^
Daily working time (hours) *	14.3 ± 2.9	r = 0.331	<0.001 ^a^	r = 0.177	0.033 ^a^
Rest time during work (minutes) **	120 (60–180)	r_s_ = −0.278	<0.001 ^d^	r_s_ = −0.194	0.019 ^d^
Work schedule			0.285 ^c^		0.289 ^c^
Morning/Afternoon	47 (32.2)	81.7 ± 19.6		72.7 ± 27.3	
Night/Day, varying according to the agricultural/fishing schedule	99 (67.8)	85.1 ± 14.8		77.6 ± 23.0	
Has another job in addition to agriculture/fishing			0.347 ^c^		0.160 ^c^
Yes ^ɫ^	7 (4.8)	89.8 ± 10.6		88.8 ± 15.8	
No	139 (95.2)	83.7 ± 16.7		75.4 ± 24.7	
Employment status			<0.001 ^c^		0.013 ^c^
Self-employed	142 (97.3)	84.8 ± 15.8		76.9 ± 23.9	
Employed	4 (2.7)	57.1 ± 20.6		46.1 ± 31.5	

^a^ Pearson’s correlation coefficient; ^b^ analysis of variance (ANOVA); ^c^ Student’s *t*-test; ^d^ Spearman’s correlation coefficient; * reported as mean ± SD; ** reported as median (25th–75th percentiles); ^#^ significantly different from farmers (*p* < 0.05); ^ɫ^ other jobs: commerce, civil construction, and construction work.

**Table 4:** Multivariable linear regression models for variables associated with workload related to agriculture/fishing and workload related to family involvement in the household (n = 146).

Variable	b (95% CI)	β	*p*	R^2^	Adjusted R^2^
**Workload related to agriculture/fishing**				19.6%	17.3%
Daily working time, hours	1.69 (0.82; 2.56)	0.298	<0.001		
Rest time during work, minutes	−0.05 (−0.08; −0.01)	−0.200	0.011		
Monthly income up to 2 minimum wages	7.18 (−0.90; 15.30)	0.140	0.081		
Self-reported White skin color/race	7.24 (−0.38; 14.90)	0.144	0.062		
**Workload related to family involvement in the household**				8.0%	6.7%
Monthly income up to 2 minimum wages	19.90 (7.78; 32.10)	0.262	0.001		
Self-reported White skin color/race	10.80 (−1.12; 22.60)	0.145	0.076		

Note: b = unstandardized regression coefficient; 95% CI = 95% confidence interval; β = standardized regression coefficient; R^2^ = coefficient of determination. Final models were obtained using backward selection, retaining variables with *p* < 0.10. Statistical significance was interpreted at *p* ≤ 0.05. Employment relationship was not entered into the main multivariable models because of its highly asymmetric distribution in the sample, with only four formally employed workers.

## Data Availability

The data presented in this study are not publicly available due to privacy and ethical restrictions related to research involving human participants. Data may be available from the corresponding author upon reasonable request.

## Supplementary Materials

The following supporting information can be downloaded at: https://www.mdpi.com/article/10.3390/nursrep16070247/s1, Supplementary File S1: STROBE Statement—Checklist of items that should be included in reports of cross-sectional studies; Table S1: Candidate variables entered in the initial multivariable linear regression models and retained or excluded after backward selection.

## Public Involvement Statement

No public involvement occurred in the design, conduct, analysis, interpretation, or reporting of this research. The rural and fishing workers participated by responding to the research instruments.

## Guidelines and Standards Statement

This manuscript was drafted according to the Strengthening the Reporting of Observational Studies in Epidemiology (STROBE) guidelines.

## Use of Artificial Intelligence

AI-assisted tools were used to support language translation, language editing, grammar checking, and the schematic design of Figure 1. Specifically, ChatGPT, based on GPT-5.5 Thinking by OpenAI, was used to support translation, language editing, and the schematic organization of Figure 1, and Grammarly Pro, version 1.2.277.1921 was used for grammar checking. These tools were not used for statistical analysis, data interpretation, definition of results, development of conclusions, or scientific decision-making. The authors reviewed and take full responsibility for all manuscript content.

## Abbreviations

The following abbreviations are used in this manuscript:

PHC	Primary Health Care
PNAB	National Primary Care Policy
PNSTT	National Policy on Workers’ Health
NASA-TLX	National Aeronautics and Space Administration Task Load Index
