# Supplementary material for: Dual Workload Related to Agriculture/Fishing and Family Involvement in the Household Among Rural and Small-Scale Fishing Workers in Southern Brazil: Implications for Nursing Care Organization in Primary Health Care
Source: Nurs Rep. 2026 Jul 15;16(7):247. doi: 10.3390/nursrep16070247 (PMC13415098; doi:10.3390/nursrep16070247)
Supplement: Supplementary file 1 [file nursrep-16-00247-s001.zip › Supplementary File S1_STROBE_Checklist.pdf]

Manuscript Title: Dual Workload Related to Agriculture/Fishing and Family Involvement in the Household among Rural and Small-Scale Fishing Workers in Southern Brazil: Implications for Nursing Care Organization in Primary Health Care

STROBE Statement—Checklist of items that should be included in reports of ***cross-sectional studies***

	**Item No**	**Recommendation**	Reported on (section/location)	Page(s)
**Title and abstract**	1	(*a*) Indicate the study's design with a commonly used term in the title or the abstract	Title and Abstract: title; Abstract—Methods	p. 1
		(*b*) Provide in the abstract an informative and balanced summary of what was done and what was found	Abstract: Background/Objectives, Methods, Results, Conclusions	p. 1
**Introduction**				
Background/rationale	2	Explain the scientific background and rationale for the investigation being reported	Introduction: scientific background on PHC, rural PHC, nursing, workers' health, decent work, family farming/small-scale fishing, NASA-TLX, and study rationale	pp. 2–5
Objectives	3	State specific objectives, including any prespecified hypotheses	Introduction: final paragraph with specific objective	p. 5
**Methods**				
Study design	4	Present key elements of study design early in the paper	Methods, Section 2.1 Study design	p. 5
Setting	5	Describe the setting, locations, and relevant dates, including periods of recruitment, exposure, follow-up, and data collection	Methods, Section 2.2 Linkage to the research project and study setting; Section 2.4 Data collection procedures	pp. 5–7
Participants	6	(*a*) Give the eligibility criteria, and the sources and methods of selection of participants	Methods, Section 2.3 Study population, eligibility criteria, and sampling	p. 6
Variables	7	Clearly define all outcomes, exposures, predictors, potential confounders, and effect modifiers.	Methods, Sections 2.1, 2.5.1, 2.5.2, and 2.6; outcomes, independent variables, NASA-TLX domains, and analytical variables defined	pp. 5, 7–10
Data sources/ measurement	8*	For each variable of interest, give sources of data and details of methods of assessment	Methods, Sections 2.4, 2.5.1 and 2.5.2; data	pp. 7–10

		(measurement). Describe comparability of assessment methods if there is more than one group	sources and measurement procedures for the structured questionnaire and two separate NASA-TLX applications	
Bias	9	Describe any efforts to address potential sources of bias	Methods, Sections 2.3, 2.4, 2.5.2, and 2.6; eligibility confirmation by self-report, interviewer training, standardized administration, interviewer-completed instruments, procedures to avoid missing data, contextual use of NASA-TLX for family involvement, and exclusion of employment relationship from main models due to highly asymmetric distribution	pp. 6–10
Study size	10	Explain how the study size was arrived at	Methods, Section 2.3; consecutive convenience sampling; 146 eligible and accessible workers included in the final analytical sample	p. 6
Quantitative variables	11	Explain how quantitative variables were handled in the analyses. If applicable, describe which groupings were chosen and why	Methods, Sections 2.5.2 and 2.6; NASA-TLX score standardization on a 0–100 scale, fixed-range workload categories, and use of continuous overall scores in multivariable analyses; Results, Table 1, Table 2, and Figure 2	pp. 9–13
Statistical methods	12	(*a*) Describe all statistical methods, including those used to control for confounding	Methods, Section 2.6 Statistical analysis; descriptive analyses, paired comparison, bivariate analyses, multivariable linear regression, criteria for model entry and retention, VIF/tolerance,	pp. 9–11

			residual plots, R^2 and adjusted R^2	
		(*b*) Describe any methods used to examine subgroups and interactions	Not applicable; no subgroup or interaction analyses were conducted. Sex was analyzed in bivariate analyses but was not significantly associated with either workload outcome	pp. 9–10
		(*c*) Explain how missing data were addressed	Methods, Sections 2.4 and 2.6; no missing data were identified for the variables analyzed in the final analytical sample. Instruments were completed by trained interviewers and checked for completeness under supervision; all analyses were conducted with n = 146	pp. 7,9-10
		(*d*) If applicable, describe analytical methods taking account of sampling strategy	Methods, Sections 2.3 and 2.6; non-probabilistic consecutive convenience sampling described, and findings interpreted as context-specific	pp. 6, 10
		(*e*) Describe any sensitivity analyses	No separate formal sensitivity analysis was conducted. A conservative analytical decision was adopted by excluding employment relationship from the main multivariable models due to its highly asymmetric distribution. The complete set of candidate variables and their retention or exclusion after backward selection is presented in Supplementary Table S1. The possible ceiling effect was examined descriptively and discussed as a limitation	pp. 10–11, 18–19; Supplementary Table S1

**Results**				
Participants	13*	(a) Report numbers of individuals at each stage of study—eg numbers potentially eligible, examined for eligibility, confirmed eligible, included in the study, completing follow-up, and analysed	Methods, Section 2.3 and Results opening paragraph; field approach, closed-household records, refusals, incomplete interview, final analytical sample, and 146 participants analyzed	pp. 6, 12
		(b) Give reasons for non-participation at each stage	Methods, Section 2.3; closed households revisited up to three times; 567 closed-household records registered; 41 eligible contacted workers declined; one incomplete interview excluded before database closure	pp. 6
		(c) Consider use of a flow diagram	Not used; no participant flow diagram was included because the study used consecutive convenience sampling in an accessible field population. Recruitment procedures, closed households, refusals, incomplete interview, and final sample are described in Section 2.3	p. 6
Descriptive data	14*	(a) Give characteristics of study participants (eg demographic, clinical, social) and information on exposures and potential confounders	Results opening paragraphs and Table 3; sociodemographic, family, and occupational characteristics, including age, sex, skin color/race, education, income, occupation, working time, rest time, additional work, and employment relationship	pp. 12–14
		(b) Indicate number of participants with missing data for each variable of interest	Methods, Sections 2.4 and 2.6; Results, Tables 1–4. No missing data were identified for variables analyzed in the	pp. 7, 10, 12–15

			final analytical sample; all displayed analyses were conducted with n = 146	
Outcome data	15*	Report numbers of outcome events or summary measures	Results, Table 1, Table 2, and Figure 2; overall and domain-specific perceived workload scores for agriculture/fishing and family involvement in the household, including mean, SD, median, IQR, workload categories, and paired comparison	pp. 10–11, 13–15
Main results	16	(*a*) Give unadjusted estimates and, if applicable, confounder-adjusted estimates and their precision (eg, 95% confidence interval). Make clear which confounders were adjusted for and why they were included	Results, Tables 3 and 4; unadjusted bivariate associations in Table 3 and adjusted multivariable models in Table 4, including b, 95% CI, β, p values, R^2, and adjusted R^2. Methods, Section 2.6 explains model construction and variables considered for entry	pp. 10-11, 13–15
		(*b*) Report category boundaries when continuous variables were categorized	Methods, Sections 2.5.2 and 2.6; category boundaries for workload levels; Results, Table 1 and Figure 2	pp. 9–13
		(*c*) If relevant, consider translating estimates of relative risk into absolute risk for a meaningful time period	Not applicable; the study used linear regression and did not estimate relative risks	pp. 13–15
Other analyses	17	Report other analyses done—eg analyses of subgroups and interactions, and sensitivity analyses	Methods, Sections 2.5.2 and 2.6; Results, Table 2; Discussion, Section 4.4. Other analyses included internal consistency of the two NASA-TLX applications, paired comparison between workload dimensions, descriptive assessment of possible ceiling effect, VIF/tolerance, residual	pp. 9–15, 18–19; Supplementary Table S1

			plot assessment, and Supplementary Table S1	
**Discussion**				
Key results	18	Summarise key results with reference to study objectives	Discussion opening paragraph and Sections 4.1 and 4.2; Conclusions. Key results summarized in relation to the study objectives	pp. 15–18, 20
Limitations	19	Discuss limitations of the study, taking into account sources of potential bias or imprecision. Discuss both direction and magnitude of any potential bias	Discussion, Section 4.4 Limitations, Strengths, and Future Research; limitations include cross-sectional design, non-probabilistic sampling, context-specific sample, two-month data collection, possible ceiling effect, unmeasured variables, gendered distribution not examined in depth, contextual use of NASA-TLX for family involvement, and no prior feasibility/acceptability assessment with PHC teams	pp. 18–20
Interpretation	20	Give a cautious overall interpretation of results considering objectives, limitations, multiplicity of analyses, results from similar studies, and other relevant evidence	Discussion, Sections 4.1–4.4; Conclusions. Cautious interpretation considering objectives, limitations, possible ceiling effect, limited explanatory power, unmeasured variables, context-specific sample, and relevant literature	pp. 15–20
Generalisability	21	Discuss the generalisability (external validity) of the study results	Methods, Section 2.3; Discussion, Section 4.4; Conclusions. Findings interpreted as context-specific and not directly generalizable to all agricultural or fishing workers, especially populations with	pp. 6, 18–20

			different employment, racial/ethnic, socioeconomic, or territorial profiles	
**Other information**				
Funding	22	Give the source of funding and the role of the funders for the present study and, if applicable, for the original study on which the present article is based	Methods, Section 2.2; Funding statement. Funding reported as CNPq Call No. 09/2023—Research Productivity Fellowships (PQ–1A), process No. 316678/2023-6	pp. 5, 20

*Give information separately for exposed and unexposed groups.

Note: An Explanation and Elaboration article discusses each checklist item and gives methodological background and published examples of transparent reporting. The STROBE checklist is best used in conjunction with this article (freely available on the Web sites of PLoS Medicine at http://www.plosmedicine.org/, Annals of Internal Medicine at http://www.annals.org/, and Epidemiology at http://www.epidem.com/). Information on the STROBE Initiative is available at www.strobe-statement.org.
