# Supplementary material for: Dual Workload Related to Agriculture/Fishing and Family Involvement in the Household Among Rural and Small-Scale Fishing Workers in Southern Brazil: Implications for Nursing Care Organization in Primary Health Care
Source: Nurs Rep. 2026 Jul 15;16(7):247. doi: 10.3390/nursrep16070247 (PMC13415098; doi:10.3390/nursrep16070247)
Supplement: Supplementary file 1 [file nursrep-16-00247-s001.zip › Table S1_Multivariable_Regression_Candidate_Variables.pdf]

Supplementary Material Table S1. Candidate variables entered in the multivariable linear regression models and retained or excluded after backward selection.

Table S1. Candidate variables entered in the initial multivariable linear regression models and retained or excluded after backward selection.

Variables	b (95% CI)	β	p	R^2
**Workload related to agriculture/fishing**				21.6%
Daily working time, hours *	1.69 (0.82; 2.56)	0.298	<0.001	
Rest time during work, minutes *	-0.05 (-0.08; -0.01)	-0.200	0.011	
Monthly income up to 2 minimum wages *	7.18 (-0.90; 15.30)	0.140	0.081	
Self-reported White skin color/race *	7.24 (-0.38; 14.90)	0.144	0.062	
Educational level up to elementary school **	1.05 (-5.92; 8.03)	0.025	0.766	
Occupation **				
Farmer	0.00	—	—	
Fisher	3.56 (-4.31; 11.40)	0.099	0.372	
Both farming and fishing	1.60 (-8.74; 11.90)	0.032	0.760	
Length of employment in agriculture/fishing, years **	0.09 (-0.07; 0.24)	0.086	0.265	
Weekly working hours **	-0.02 (-0.16; 0.11)	-0.028	0.754	
**Workload related to family involvement in the household**				15.1%
Monthly income up to 2 minimum wages *	19.90 (7.78; 32.10)	0.262	0.001	
Self-reported White skin color/race *	10.80 (-1.12; 22.60)	0.145	0.076	
Occupation **				
Farmer	0.00	—	—	
Fisher	3.91 (-8.18; 16.00)	0.073	0.524	
Both farming and fishing	12.00 (-3.90; 27.90)	0.161	0.138	
Length of employment in agriculture/fishing, years **	0.08 (-0.17; 0.32)	0.051	0.538	
Daily working time, hours **	1.04 (-0.32; 2.39)	0.123	0.134	
Rest time during work, minutes **	-0.01 (-0.07; 0.04)	-0.039	0.639	
Another job in addition to agriculture/fishing **	10.10 (-8.00; 28.20)	0.089	0.272	

Note: b = unstandardized regression coefficient; 95% CI = 95% confidence interval; β = standardized regression coefficient; R^2 = coefficient of determination. * Variables retained in the final model after backward selection. ** Variables excluded from the final model after backward selection. Employment relationship was not entered into multivariable models because of its highly asymmetric distribution in the sample.
